# Dietary Antioxidants and Cardiovascular Health—Editorial Comments and Summary

**DOI:** 10.3390/antiox12081598

**Published:** 2023-08-11

**Authors:** Magdalena Kwaśniewska, Anna Waśkiewicz, Wojciech Drygas

**Affiliations:** 1 Department of Social and Preventive Medicine, Faculty of Health Sciences, Medical University of Lodz, 90-752 Lodz, Poland; 2 Department of Epidemiology, Cardiovascular Disease Prevention and Health Promotion, National Institute of Cardiology, 04-628 Warsaw, Poland; awaskiewicz@ikard.pl (A.W.); wdrygas@ikard.pl (W.D.); 3 World Institute of Family Health, Calisia University, 62-800 Kalisz, Poland

The influence of dietary antioxidants on health is supported by a large body of evidence. The adequate intake of polyphenols, antioxidant vitamins, and minerals may contribute to better clinical outcomes, including morbidity, mortality, and quality of life. These outcomes seem particularly important in the context of cardiovascular diseases (CVDs), which are the leading cause of mortality worldwide. 

Therefore, this Special Issue of *Antioxidants,* entitled “Dietary Antioxidants and Cardiovascular Health”, collates 15 publications: 13 original manuscripts and 2 review papers. The contributors provide novel and comprehensive insights into the effects of the intake of well-known nutrients, as well as those with potential antioxidant properties. The presented findings are related to various aspects of CVD: the relationship between the dietary intake of particular nutrients and the function of myocardial tissue, the plasma and urinary profiles of CVD risk factors, and epidemiological studies investigating the effects of nutrition models on cardiac outcomes in adults and adolescents. 

In their study, Sutanto et al. [1] aimed to explore the effects of naringenin, a grapefruit flavonoid, on cardiomyocyte action using a detailed in silico model of ventricular electrophysiology. The obtained findings demonstrate that naringenin significantly prolongs the duration of cardiomyocyte action in a concentration-dependent manner, with a greater effect in females than in males. Therefore, this suggests that this flavonoid has a potentially higher proarrhythmic risk in women. Cardiac function may be also modified by the inappropriate intake of zinc, one of the most important minerals with antioxidant properties. According to the study by Szadkowska et al. [2], dietary zinc plays an important role in enhancing pathways of myocardial tissue repair in elderly individuals. The authors found poorer cardiac function in elderly individuals with lower zinc intake and highlighted the essential need for proper zinc consumption in this age group. Aside from zinc intake, the deficiency of vitamin C may also affect coronary artery vasomotor function and the development of coronary atherosclerotic plaque. Although vitamin C is a well-known modulator of CVD risk, functional evidence supporting a causal relationship is rather scarce. Skovsted et al. [3] demonstrated that poor vitamin C status exacerbated dysfunction of atherosclerotic coronary arteries negatively affects coronary artery function, especially in association with a high-fat diet and chronic dyslipidaemia. 

Several other studies in this Special Issue support the potential role of polyphenols as potent antioxidants. An original study by Olivares-Caro et al. [4] revealed that the regular consumption of *Berberis microphylla* G. Frost (calafate) substantially reduced the plasma concentration of some CVD markers such as thrombomodulin, adiponectin, sE-selectin, sICAM-1, and proMMP-9. These changes suggest that calafate has cardioprotective effects when ingested as part of a high-fat diet. Interestingly, overweight/obese women may benefit from drinking apple/berry juice as a part of a healthy lifestyle in order to reduce the risk of CVD. According to the findings of Habanova et al. [5], a daily intake of 300 mL of the apple/berry juice for 6 weeks significantly improved lipid profiles, lipoprotein subfractions, antioxidant status, and the concentration of glucose and magnesium in middle-aged women with body mass index values of ≥25 kg/m^2^. The vascular health effects of quercetin, a well-known monomeric polyphenol, are comprehensively discussed in a review paper by Terao [6]. The author focuses on the bioavailability and bioefficacy of quercetin glycosides as this form of quercetin is exclusively present in plant-based foods. Several studies support the beneficial role of quercetin and glycoside-rich foods in terms of CVD health and potential clinical applications. 

Fermented beverages rich in natural polyphenols, such as beer or wine, are known for their protective effects against oxidative stress. A study by Gallinat et al. [7] provides insights into the potential mechanisms through which beer intake might affect the response of the heart to oxidative damage. The authors report that beer consumption had dose-dependent effects on the expression of genes related to the electron transport chain and spliceosome, as well as dose-specific effects on inflammation and immune response. 

Studies investigating the intake of dietary antioxidants in the context of hypertension reveal several important findings. Lopez-Moreno et al. [8] revealed the in vivo antioxidant effect of red quinoa hydrolysate, its ability to ameliorate hypertension, and its associated complications. Blood pressure can be significantly reduced by the appropriate intake of several herbal supplements. A meta-analysis of 31 studies performed by Lipert et al. [9] demonstrated that resveratrol, cherry and beetroot juice, bergamot extracts, barberry, and pycnogenol significantly lowered both systolic and diastolic blood pressure compared with a placebo. Therefore, the consumption of the abovementioned nutrients and food items can be considered part of a successful antihypertensive strategy and cardioprotection. Interesting results are also presented by Ramirez-Garza et al. [10], who investigated the association between nitric oxide and CVD risk among adolescents. The authors found that urinary nitric oxide levels were positively correlated with the consumption of fruits and vegetables rich in polyphenols and negatively correlated with systolic blood pressure. These findings are significant as they reinforce existing recommendations on appropriate dietary choices in health promotion and CVD prevention. Additionally, Laveriano-Santos et al. [11] aimed to develop and validate a method to identify and quantify urinary microbial phenolic metabolites, exploring the relationship between these metabolites and dietary polyphenols in Spanish adolescents. This new method turned out to be accurate and suitable for the quantification of phenolic metabolites in large epidemiological studies.

The findings of the study by Gawron-Skarbek et al. [12] broaden our current knowledge on the role of fatty acids in CVD protection in older individuals. The authors aimed to assess the impact of fatty acid intake on the total antioxidant capacity of plasma and the salivary C-reactive protein. The obtained findings show that the anti-inflammatory and antioxidant impacts of dietary saturated and unsaturated fatty acids are related to vitamin C intake. 

Another important study was conducted by Barragan et al. [13] on the role of trace elements in CVD risk. According to the available literature, trace elements can have both antioxidant and pro-oxidant properties. This population study, which recruited 484 participants aged 18–80 years from the Mediterranean region, revealed that the combined effect of higher plasma trace element levels (mainly Zn, Cu and Se) is directly associated with elevated plasma lipids, whereas the mixture effect in urine is primarily associated with plasma glucose. Therefore, increased trace element exposures should be considered with caution.

The two last articles in this Special Issue are large-population studies that focus on the impact of dietary patterns and the intake of certain food groups on CVD risk in Polish participants [14,15]. Szypowska et al. [14] assessed the inflammatory potential of the diets of participants enrolled in the Polish arm of the Prospective Urban and Rural Epidemiological (PURE) study, with a special focus on determining the correlation between dietary inflammatory index score and dietary content as well as CVD risk factors. The obtained results demonstrate more favourable CVD outcomes among individuals following diets rich in plant-based food. In order to achieve beneficial anthropometric and biochemical CVD indices, pro-inflammatory items such as refined food, red meat, dairy (especially high-fat dairy), sweets, juices, alcohol, potatoes, sugar, honey, French fries, fried fish, and processed/high-fat poultry should be eliminated from diets. 

Finally, a study of dietary antioxidant intake and CVD characteristics in participants following different nutrition patterns was conducted by Kwasniewska et al. [15]. An analysis was performed in the representative sample of 13,318 adults participating in the National Multicentre Health Surveys (WOBASZ). Taking into account the growing popularity of diets containing fewer animal products, the authors assessed the intake of antioxidants, quality of nutrition and CVD risk among vegetarians, flexitarians, and omnivores. Surprisingly, flexitarians did not have a better quality of nutrition, higher antioxidant intake, or CVD profile compared with omnivores. The vegetarian study group had more favourable cardiometabolic profiles but was too small to draw substantial conclusions. Importantly, the authors conclude that, regardless of the type of a diet, strategies must be developed to provide Polish adults with guidance on a properly balanced diet.

We would like to thank all authors for submitting papers to this Special Issue as well as the reviewers for their commitment throughout the publication process.

## References

[1] Sutanto H., Hertanto D.M., Susilo H., Wungu C.D.K. (2022). Grapefruit Flavonoid Naringenin Sex-Dependently Modulates Action Potential in an In Silico Human Ventricular Cardiomyocyte Model. Antioxidants.

[2] Szadkowska I., Kostka T., Wlazeł R.N., Kroc, Jegier A., Guligowska A. (2023). Dietary Zinc Is Associated with Cardiac Function in the Older Adult Population. Antioxidants.

[3] Skovsted G.F., Skat-Rørdam J., Frøkiær A.P., Jensen H.E., Tveden-Nyborg P., Lykkesfeldt J. (2022). Vitamin C Deficiency Exacerbates Dysfunction of Atherosclerotic Coronary Arteries in Guinea Pigs Fed a High-Fat Diet. Antioxidants.

[4] Olivares-Caro L., Nova-Baza D., Radojkovic C., Bustamante L., Duran D., Mennickent D., Melin V., Contreras D., Perez A.J., Mardones C. (2023). *Berberis microphylla* G. Forst Intake Reduces the Cardiovascular Disease Plasmatic Markers Associated with a High-Fat Diet in a Mice Model. Antioxidants.

[5] Habanova M., Holovicova M., Scepankova H., Lorkova M., Gazo J., Gazarova M., Pinto C.A., Saraiva J.A., Estevinho L.M. (2022). Modulation of Lipid Profile and Lipoprotein Subfractions in Overweight/Obese Women at Risk of Cardiovascular Diseases through the Consumption of Apple/Berry Juice. Antioxidants.

[6] Terao J. (2023). Potential Role of Quercetin Glycosides as Anti-Atherosclerotic Food-Derived Factors for Human Health. Antioxidants.

[7] Gallinat A., Vilahur G., Padro T., Badimon L. (2023). Effects of Antioxidants in Fermented Beverages in Tissue Transcriptomics: Effect of Beer Intake on Myocardial Tissue after Oxidative Injury. Antioxidants.

[8] López-Moreno M., Jiménez-Moreno E., Gallego A.M., Pasamontes G.V., Ocio J.A.U., Garcés-Rimón M., Miguel-Castro M. (2023). Red Quinoa Hydrolysates with Antioxidant Properties Improve Cardiovascular Health in Spontaneously Hypertensive Rats. Antioxidants.

[9] Lipert A., Szadkowska I., Matusiak-Wieczorek E., Kochan E. (2022). The Effect of Herbal Supplements on Blood Pressure: Systematic Review and Meta-Analysis. Antioxidants.

[10] Ramírez-Garza S.L., Laveriano-Santos E.P., Arancibia-Riveros C., Carrasco-Jimenez J.C., Bodega P., de Cos-Gandoy A., de Miguel M., Santos-Beneit G., Fernández-Alvira J.M., Fernández-Jiménez R. (2022). Urinary Nitric Oxide Levels Are Associated with Blood Pressure, Fruit and Vegetable Intake and Total Polyphenol Excretion in Adolescents from the SI! Program. Antioxidants.

[11] Laveriano-Santos E.P., Marhuenda-Muñoz M., Vallverdú-Queralt A., Martínez-Huélamo M., Tresserra-Rimbau A., Miliarakis E., Arancibia-Riveros C., Jáuregui O., Ruiz-León A.M., Castro-Baquero S. (2022). Identification and Quantification of Urinary Microbial Phenolic Metabolites by HPLC-ESI-LTQ-Orbitrap-HRMS and Their Relationship with Dietary Polyphenols in Adolescents. Antioxidants.

[12] Gawron-Skarbek A., Guligowska A., Prymont-Przymińska A., Nowak D., Kostka T. (2023). The Anti-Inflammatory and Antioxidant Impact of Dietary Fatty Acids in Cardiovascular Protection in Older Adults May Be Related to Vitamin C Intake. Antioxidants.

[13] Barragán R., Sánchez-González C., Aranda P., Sorlí J.V., Asensio E.M., Portolés O., Ortega-Azorín C., Villamil L.V., Coltell O., Llopis J. (2022). Single and Combined Associations of Plasma and Urine Essential Trace Elements (Zn, Cu, Se, and Mn) with Cardiovascular Risk Factors in a Mediterranean Population. Antioxidants.

[14] Szypowska A., Regulska-Ilow B., Zatońska K., Szuba A. (2023). Comparison of Intake of Food Groups Based on Dietary Inflammatory Index (DII) and Cardiovascular Risk Factors in the Middle-Age Population of Lower Silesia: Results of the PURE Poland Study. Antioxidants.

[15] Kwaśniewska M., Pikala M., Grygorczuk O., Waśkiewicz A., Stepaniak U., Pająk A., Kozakiewicz K., Nadrowski P., Zdrojewski T., Puch-Walczak A. (2023). Dietary Antioxidants, Quality of Nutrition and Cardiovascular Characteristics among Omnivores, Flexitarians and Vegetarians in Poland—The Results of Multicenter National Representative Survey WOBASZ. Antioxidants.

